# Identification and evaluation of deep foundation pit construction risks based on Grey-DEMATEL-Fuzzy comprehensive evaluation method

**DOI:** 10.1371/journal.pone.0304095

**Published:** 2024-08-27

**Authors:** Hu Hao, He Jian, Liu Peiling, Zhu Cong

**Affiliations:** College of Civil Engineering, Chongqing University of Arts and Sciences, Chongqing, China; Instituto Tecnologico de Aeronautica, BRAZIL

## Abstract

In recent years, foundation pit construction has been rapidly developing in the direction of deep and large-scale, leading to the frequent occurrence of construction accidents. The pit construction process is characterised by a complex environment, high construction risk, and numerous coupling effects between the construction risk factors. In this paper, 23 main accident-causing factors in foundation pit construction are determined based on the six major risk accident types. In addition, the Grey-DEMATEL-Fuzzy comprehensive evaluation method of the risk evaluation model is introduced for better prediction and judgment of risk level, which combines the grey system theory with the method of decision-making experimental analysis, and in the case of inaccurate or incomplete information, the use of less data can achieve the evaluation results with a high degree of reliability, and it will effectively avoid the impact of the lack of information as well as the subjectivity in the process of risk evaluation. Through the Grey-DEMATEL method, the central degree value for each risk indicator factor is calculated, the coupling role and importance of each risk indicator are analysed, and the indicator weights are calculated. Based on the calculated weights, the fuzzy comprehensive evaluation method is used to evaluate the overall risk level. The empirical research on the deep foundation pit construction project of Haitangxi subway station in Chongqing reveals that the excessive lateral earth pressure on the pile wall is the most prominent risk factor. The overall risk level of the construction process is medium, and the risk is within the controllable range. On this basis, corresponding preventive measures can be formulated, providing a basis for risk prevention in the construction of deep foundation pit projects.

## Introduction

With sustained and rapid economic and social development and higher demands for quality of life, the global urbanisation process and infrastructure projects have been vigorously developed, with an increasing trend in the scale and number of constructions. According to the "Transportation Statistics Annual Report 2022", the U.S. road system had about 4.2 million centerline miles and 8.8 million lane-miles in 2020 (essentially the same as in 2018), with more than 618,400 bridges (an increase of 0.4% from 2018) [1]. According to "2022 Inland Transport Statistics for Europe and North America", infrastructure investment in 56 member countries of UNECE is on an upward trend between 2018–2020 [2].

According to the statistics of the Ministry of Transport of the People’s Republic of China, a total of 292 urban railway lines have been opened and operated in 54 cities as of May 2023, with an operating length of 9,652.6 km. The actual number of trains in operation is 3.22 million, with 2.49 billion passenger trips, including 1.5 billion inbound passenger trips [3]. The "2021 Bulletin of China’s Urban Construction Situation" indicates that by the end of 2021, the built-up area of cities nationwide covered an area of 62,400 km^2^, a year-on-year increase of 2.80%. Investment in fixed assets in urban facilities nationwide amounted to 2.75 trillion CNY, a year-on-year increase of 4.93%. During the period from 2012–2021, the urban built-up area in China reached 54,800 km^2^ on average per year, and the annual investment in basic implementation reached about 2.22 trillion yuan, exhibiting an increasing trend year by year [4].

As an important resource for urban development, underground space has received increasing attention since the 1990s. By the end of 2021, the total length of completed metro lines reached 7742.43 km, of which 6,192.96 km were underground lines [5]. With the significant development of underground engineering, the exploitation of urban underground space has gradually advanced to deeper underground, and the scale, depth and difficulty of the project have dramatically increased. For example, the deep excavation pit depth of the Shanghai Metro Line 4 restoration project is nearly 41 m, the depth of the Shanghai World Expo substation foundation pit is nearly 34 m, the maximum excavation depth of the Tianjin Station Traffic Hub project is 33.5 m, the maximum excavation depth of the Tianjin Cultural Centre foundation pit is nearly 30 m, and the maximum excavation depth of the Tianjin Chow Tai Fook foundation pit is more than 30 m. As a result, accidents in underground projects have become more frequent. In 2013, a landslide occurred at the construction site of an underground station in Chaoyang District, Beijing, resulting in five deaths. In 2016, a collapse occurred at the pit construction site for a commercial complex in Pudong New District, Shanghai, resulting in one fatality. In 2017, a collapse occurred at a pit construction site for a subway station in Tianhe District, Guangzhou City, resulting in seven fatalities. In 2018, a fire accident happened at a pit construction site in Manhattan, New York, the United States, resulting in one death and three injuries. In 2020, a collapse accident at a subway station pit construction site in Nanjing, Jiangsu Province, China, killed three people. All these accidents indicate that adequate attention must be paid to the risk factors in deep foundation pit construction. Therefore, it is necessary to identify and assess the risks of deep excavation in order to prevent accidents.

## Literature review

In recent years, the study of risk analysis has gradually extended to various sectors of engineering projects. In 2021, Yang proposed an innovative financial risk analysis framework to address the problem of insufficient data financial risk analysis in small and medium-sized enterprises [6]. In 2022, Gao et al. [7] addressed the issue of sustainable urban development by scientifically predicting and assessing future ecological risks under different scenarios. In order to conduct a synthetic climate risk analysis for Earth’s forests in the 21st century, Anderegg et al. [8] combined outputs from multiple mechanistic and empirical approaches to modelling carbon, biodiversity, and disturbance risks. In 2023, Tang et al. [9] proposed a new risk prioritisation model based on the belief Jensen-Shannon divergence measure and Deng entropy for the thin plate steel production process. In order to improve the accuracy and reliability of FMEA risk analysis, a new FMEA model based on Dempster Shafer evidence theory (DST) and improved logistic probability transformation function in the grey relational projection method (GRPM) was also proposed for aircraft turbine rotor blades and steel production processes [10]. For shipborne medium-voltage DC power supply systems, Hua et al. [11] proposed an improved risk priority ranking method employing heterogeneous information expression structures (i.e., critical numbers, interval numbers, triangular fuzzy numbers, and linear Z-numbers).

However, few studies have investigated the risk of the foundation pit project. McFeat-Smith [12] analysed and statistically evaluated the risks during underground construction using the IMS system. A total of 15 risk categories and 33 risk types were summarised. Van Staveren et al. [13] suggested that factors such as the geology of the foundation pit project, water quality, and precipitation have a significant effect on the construction safety of the deep foundation pit. Based on WBS-RBS, Zhou et al. [14] constructed a comprehensive risk factor identification model for deep foundation pit construction and built a risk assessment index system. Pan et al. [15] applied the mutation level method to the risk assessment of deep foundation pit construction in subway stations. Risk items that can easily be overlooked were highlighted, providing an effective way to alert managers about risk terms. Phoon et al. [16] summarised and analysed the existing general risk evaluation methods. The corresponding risk acceptance criteria were established and divided into three types, namely parametric risk, management risk, and model risk. Chen et al. [17] evaluated the deep foundation pit construction of a metro station in Chongqing. Combined with the deep foundation pit construction project of Shishan Park Station of Nanning Railway Transit Line 5, Mong et al. [18] used the WBS-RBS method to comprehensively identify the risk indicators from three aspects, i.e., excavation, support system, and enclosure structure system.

These studies have further promoted the development of urban deep foundation pit engineering and facilitated the urbanisation process. However, there is still a lack of systematic and scientific research on the risk management of deep foundation pit construction, and the evaluation method suffers from the problems of high subjectivity and unreliable data. Therefore, this paper adopts the Grey-DEMATEL model to analyse and evaluate the risk for specific cases of deep foundation pit construction. The centrality-causality diagram is obtained based on the in-depth analysis of the interactions between risk indicators, significantly reducing the influence of human subjective factors and environmental uncertainty. Furthermore, a fuzzy comprehensive evaluation method is adopted to predict the overall risk, thereby determining the risk level and providing corresponding countermeasures.

## Methodologies

### Deep foundation pit construction risk influencing factor identification

For the potential risk factors existing in the construction of the deep foundation pit, relevant studies were reviewed [12–21], and expert consultation was conducted. On this basis, six major risks during deep foundation pit construction were derived, including borehole wall collapse, landslide of soil in the pit, leakage from enclosure structure, support instability, enclosure kick damage, and surging or spillage at the bottom of the pit. Furthermore, according to the risk breakdown structure (RBS), the risk evaluation indexes for the deep foundation pit construction are listed in Table 1.

**Table 1:** Risk assessment indexes for deep foundation pit construction projects.

Type of incident	Cause of incident
Borehole wall collapse (R1)	Failure of mud quality (R11)
	Existence of subsurface obstructions (R12)
	Excessive lateral earth pressure on pile wall (R13)
	Verticality of trench wall not in accordance with requirements (R14)
Landslide of soil in pit (R2)	Poor geology (R21)
	Decrease in soil stability (R22)
	Excessive excavation (R23)
	Excessive stockpiling at the top of the slope (R24)
	Excessive slope steepness (R25)
Leakage from enclosure structure (R3)	The spinning pile diameter does not meet design requirements (R31)
	Precipitation not in place (R32)
	Mud trapped in joints (R33)
	Joint cracking (R34)
	Honeycombing and delamination of maintenance body (R35)
Support instability (R4)	Improper choice of support programme (R41)
	Inadequate design of enclosure structure (R42)
	Insufficient stiffness and strength of enclosure structure (R43)
	Excessive pile load at the top of the embankment (R44)
	Failure of horizontal support system (R45)
	Excessive slope steepness (R46)
	Over-excavation (R47)
Enclosure kick damage (R5)	Insufficient soil reinforcement in the pit (R51)
	Insufficient depth of enclosure in soil (R52)
	Over-excavation (R53)
	Base plate not poured in time (R54)
	Failure to design for pressurised water (R55)
Surging or spillage at the bottom of the pit (R6)	Insufficient rainfall (R61)
	Destruction of rainwater wells (R62)

### Influencing factors of deep foundation pit construction risk based on Grey-DEMATEL

#### Introduction to the Grey-DEMATAL methodology

The Decision-Making Trial and Evaluation Laboratory (DEMATEL) is a systematic method for solving complex problems proposed by Gabus and Fontela in 1971. It analyses the complex structure and causal relationship between indicators by constructing directed graphs and matrices. Constructing directed graphs and matrices to analyse the complex structure and causal relationship between indicators is widely used to identify influencing factors in complex systems. However, this method is affected by human subjective factors and environmental uncertainty [22–29]. Grey DEMATEL, on the other hand, is a method that combines grey system theory with experimental analysis for decision-making. It is possible to achieve high reliability of the evaluation results using less data in case of inaccurate or incomplete information. On this basis, using the Grey-DEMATEL method to analyse the risk influencing factors in the construction process of a deep foundation pit can reduce the uncertainty of risk factors and the subjectivity of evaluation, leading to a better prediction and judgement of the risk level [30–36].

#### Establishment of the Grey-DEMATAL risk model

*Establishment of the grey direct relationship matrix*.

The duplicate indicators were removed. In terms of the above 23 risk indicators, six experts in deep foundation pit construction were invited to compare and evaluate the interactions between the indicators according to a score of 0–4, and an initial matrix A was obtained. Among different semantic variables, "no influence = 0, very low influence = 1, low influence = 2, high influence = 3, and very high influence = 4". Based on the five-level grey semantic scale in Table 2, the initial matrix was converted to a grey number matrix $X^{1},X^{2},X^{3},X^{4},X^{5},X^{6}$ according to the grey theory.

**Table 2:** Grey semantic scale.

Semantic variable	Rating value	Corresponding grey value
No influence (N)	0	[0.00, 0.00]
Very low influence (VL)	1	[0.00, 0.25]
Low influence (L)	2	[0.25, 0.50]
High influence (H)	3	[0.50, 0.75]
Very high influence (VH)	4	[0.75, 1.00]

According to the conditions of each expert’s working unit, working years, and number of deep foundation pit projects, the expert’s knowledge of deep foundation pit construction was judged using the weight semantic scale in Table 3. The corresponding weights were given to different experts, and these weights were fuzzy.

**Table 3:** Weighted semantic scale.

Semantic variables	Not important	Less important	Important	More important	Very important
Corresponding grey numbers	[0.0, 0.3]	[0.3, 0.5]	[0.4, 0.7]	[0.5, 0.9]	[0.7, 1.0]

*Calculation of the direct impact matrix S*.

(1) The upper and lower limits of the grey value were calculated for normalisation:

$$\underline{⊕}{\tilde{X}}_{ij}^{k}=(\underline{⊕}X_{ij}^{k}-\min\underline{⊕}X_{ij}^{k})/\Delta_{\min}^{\max}$$ (1)

$$\bar{⊕}{\tilde{X}}_{ij}^{k}=(\bar{⊕}X_{ij}^{k}-\min\bar{⊕}X_{ij}^{k})/\Delta_{\min}^{\max}$$ (2)

where $\underline{⊕}X_{ij}^{k}$ and $\bar{⊕}X_{ij}^{k}$ are the lower and upper limits of the scores for *k* experts, respectively; $\underline{⊕}{\tilde{X}}_{ij}^{k}$ and $\bar{⊕}{\tilde{X}}_{ij}^{k}$ are the lower and upper limits of the standardised scores for *k* experts, respectively; $\Delta_{\min}^{\max}=\max\bar{⊕}X_{ij}^{k}-\min\bar{⊕}X_{ij}^{k}$; *i*, *j* = 1,2,3⋯,23, *k* = 1,2,⋯,6.

(2) The clarity matrix is calculated using normalised grey values:

$$Y_{ij}^{k}=\frac{\underline{⊕}{\tilde{X}}_{ij}^{k}(1-\underline{⊕}{\tilde{X}}_{ij}^{k})+(\bar{⊕}{\tilde{X}}_{ij}^{k}\times \bar{⊕}{\tilde{X}}_{ij}^{k})}{1-\underline{⊕}{\tilde{X}}_{ij}^{k}+\bar{⊕}{\tilde{X}}_{ij}^{k}}$$ (3)

(3) The standardised clarity $S_{ij}^{k}$ is calculated as follows:

$$S_{ij}^{k}=\min\underline{⊕}X_{ij}^{k}+Y_{ij}^{k}\Delta_{\min}^{\max}$$ (4)

(4) The weights *ω*_*k*_ were assigned to the normalised and clarified greyness matrix to calculate the direct influence matrix *S* = ⌊*S*_*ij*_⌋:

$$S_{ij}=\omega_{1}S_{ij}^{1}+\omega_{2}S_{ij}^{2}+\cdots +\omega_{6}S_{ij}^{6}$$ (5)

Among them, $\sum_{k=1}^{6}\omega_{k}=1$.

Calculation of the integrated impact matrix Z.

(1) The normalised direct impact matrix *N* is calculated as follows:

$$N=(1/\underset{1\leq i\leq 23}{\max}\sum_{j=1}^{23}S_{ij})•S$$ (6)

(2) Calculate the integrated impact matrix *Z*.


$$Z=N{\left(1-N\right)}^{-1}$$ (7)

*Calculation of the centrality and causality for each risk factor*.

(1) The degree of influence *f*_*i*_ and the degree of being influenced *e*_*i*_ were calculated for each risk indicator based on the combined influence matrix *Z*:

$$\{\begin{array}{c} f_{i}=\sum_{j=1}^{n}Z_{ij}\\ e_{i}=\sum_{j=1}^{n}Z_{ji} \end{array}$$ (8)

(2) The centrality *m*_*i*_ and causality *n*_*i*_ of the risk indicator were calculated from the degree of influence and the degree of being influenced:

$$\{\begin{array}{c} m_{i}=f_{i}+e_{i}\\ n_{i}=f_{i}-e_{i} \end{array}$$ (9)

*Plotting the causal distribution of centrality–causality*. Based on the results of the centrality and causality calculations, the relative position of each influencing factor was plotted on a scatterplot.

### Risk evaluation of deep foundation pit construction based on fuzzy comprehensive evaluation method

After identifying deep foundation pit construction risk indicators and analysing interactions between indicators, the influence degree of these indicators needs to be quantified. The risk system of the deep foundation pit construction has strong complexity and uncertainty, and the comprehensive influence degree of its risk is fuzzy and difficult to quantify in the evaluation process. The fuzzy comprehensive evaluation method can quantitatively analyse the fuzzy and difficult-to-quantify risk factors by considering the hierarchical nature of indicators [37–42]. Therefore, the fuzzy comprehensive evaluation method is used to analyse the influence degree of deep foundation pit construction risk.

#### Determination of risk indicator weights

In the calculation of indicator weights, the traditional fuzzy comprehensive evaluation method is often combined with the analytic hierarchy process, entropy weight method, Delphi method, and other methods to derive the weights. The indicators are independent of each other, ignoring the interaction between the factors. Therefore, this study uses the results of Grey-DEMATEL analysis to calculate the weights, i.e., the centrality value of each impact indicator is normalised to obtain the weight of each risk indicator. The calculation process can be expressed as follows:

$$W_{i}=m_{i}/\sum_{i=1}^{n}m_{i}$$ (10)

### The evaluation process of the fuzzy comprehensive evaluation method

(1) The evaluation sub-target set *U* was established as follows:

$$U=(U_{1},U_{2},\cdots ,U_{S})$$ (11)

(2) Based on the results of the calculation of the above weights, determine the set of sub-target weights *A*:

$$A=(A_{1},A_{2},\cdots ,A_{S})$$ (12)

where $0<A_{i}\leq 1,\sum_{i=1}^{s}A_{i}=1,(i=1,2,\cdots ,s)$.

(3) Determining the evaluation level of risk indicator factors.

Based on the 5-level risk impact evaluation, the evaluation level of risk impact factors of deep foundation pit construction is divided into five levels: very high, high, medium, low and very low, corresponding to the probability of occurrence of [0.8,1], [0.6,0.8), [0.4,0.6), [0.2,0.4), [0.0,0.2), i.e., $V_{i}=\{v_{1},v_{2},\cdots ,v_{5}\}=\{\left[0.8,1],\;[0.6,0.8),\;[0.4,0.6),\;[0.2,0.4),\;[0.0,0.2\right)\}$.

(4) Determining the evaluation matrix *R*_*i*_.

Experts were invited to evaluate and score each risk indicator to obtain the evaluation matrix *R*_*i*_:

$$R_{i}=[\begin{array}{cccc} r_{11} & r_{12} & \cdots & r_{1n}\\ r_{21} & r_{21} & \cdots & r_{2n}\\ \vdots & \vdots & \vdots & \vdots \\ r_{m1} & r_{m2} & \cdots & r_{mn} \end{array}]$$ (13)

(5) Calculate the combined evaluation vector *B*_*i*_ for each sub-target.

$$B_{i}=W_{i}•R_{i}$$ (14)

where *i* = 1,2,⋯,*s*.

(6) Formation of sub-target evaluation matrix quantities *B*:

$$B={\left(B_{1},B_{2},\cdots ,B_{S}\right)}^{T}$$ (15)

(7) Determining the total target score vector *C*:

C=A•B (16)

(8) From the principle of maximum affiliation, the comprehensive evaluation grade of deep foundation pit risk can be obtained.

### Risk assessment process for deep foundation pit construction

**Fig 1:**
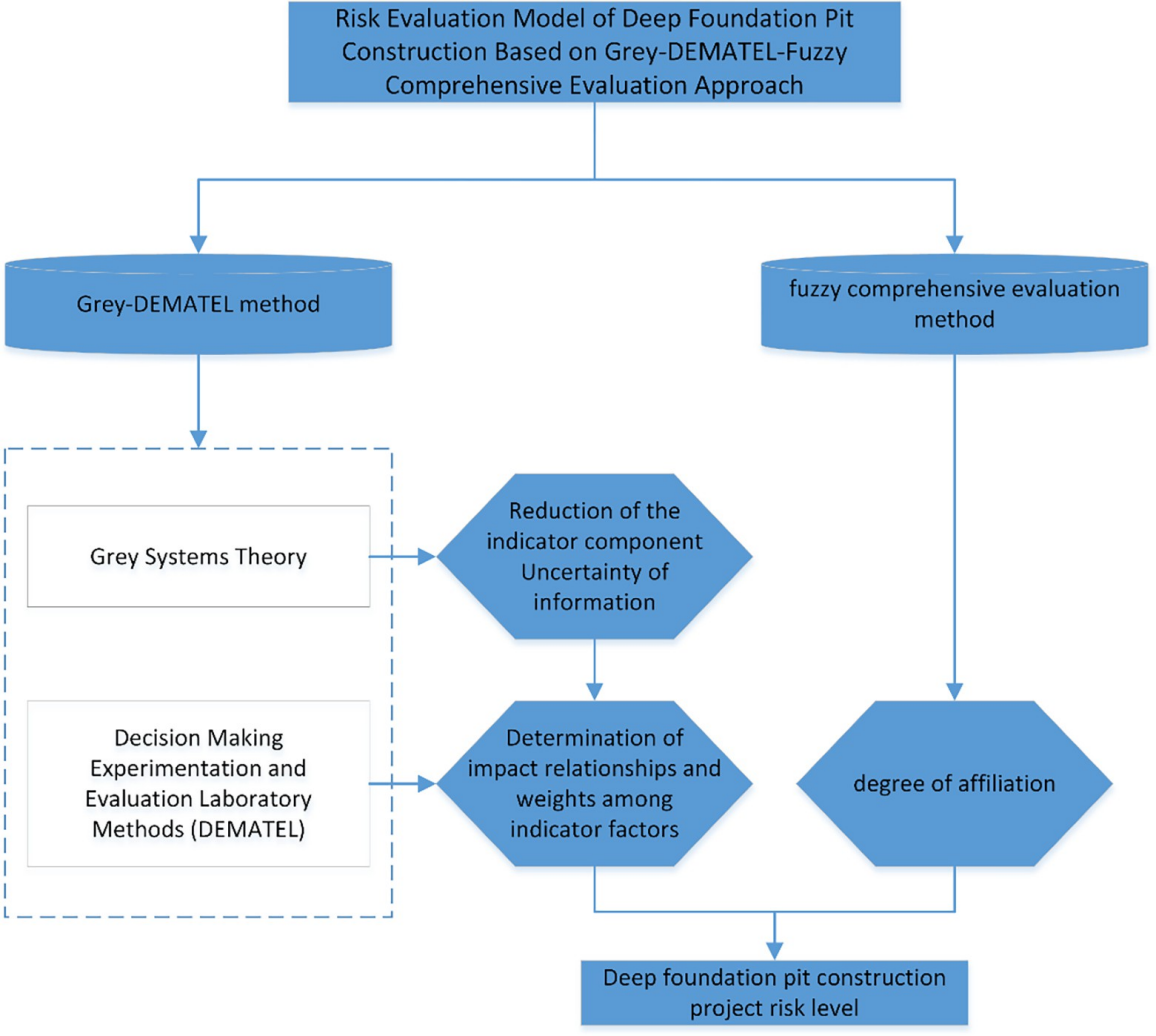
The flow chart of deep foundation pit construction risk evaluation based on the Grey-DEMATEL-Fuzzy Comprehensive Evaluation method.

In this study, the uncertain information of indicators is first quantitatively scored based on the grey system theory. Afterwards, the relationship and weight between the indicators are determined by the DEMATEL method. Finally, the affiliation value of the risk level of each indicator is calculated according to the fuzzy comprehensive method, and the risk evaluation level is obtained. The evaluation process of deep foundation pit construction risk based on the Grey-DEMATEL-Fuzzy Comprehensive Evaluation method is illustrated in Fig 1.

## Case study

### Project overview

**Fig 2:**
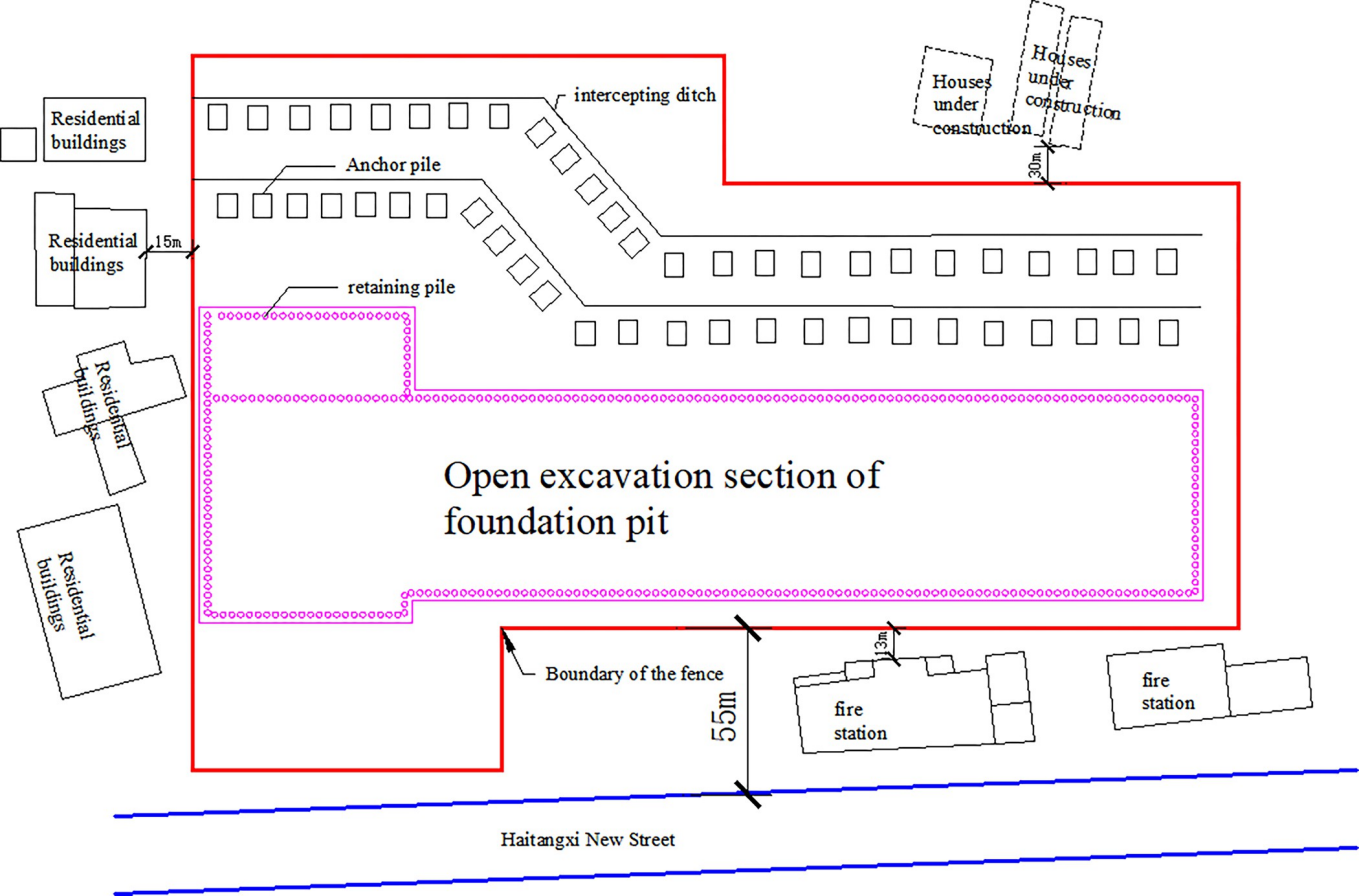
Excavation plan diagram.

Chongqing Haitangxi Metro Station is an island-type station with a half-open and half-dark scheme. The station pit is a deep pit of 139.6 m long, with an average depth of 32 m and a width of 22.8 m. All types of pipelines on the site have been relocated or removed, which does not affect the excavation of the foundation pit. The excavation plan is illustrated in Fig 2.

(1) Support situation: The safety level of the excavation pit is Grade I. The excavation method is vertical excavation, supported by row piles and the internal support system. The row piles are supported by 197 drilled piles of 1.2 m in length with a spacing of 2–35 m. The concrete strength grade is C30 and is embedded in the middle weathered rock layer for 5 m. The soil layer between the row piles and the highly weathered layer is sprayed concrete with a 100 mm thick C25 reinforcement mesh consisting of 8 @ 200 × 200 reinforcing bars. Drainage holes in the form of plum blossoms are arranged on the slope surface. Problems with the design and construction of the row piles and the internal support system, as well as in material quality, may lead to safety accidents. The diameter of the steel support is 609 mm, and the wall thickness is 16 mm.

(2) Surrounding buildings: The station is located on the site of Kerui Pharmaceutical Factory at the intersection of Longhuang Road and Haitangxi New Street. It is bounded on the north by Longhuang Road, on the west by Haitangxi New Street near the fire station, on the south by a construction site, and on the east by Kerui Pharmaceutical Factory. The entire construction site is narrow and complex, and the surrounding buildings (structures) are complex.

(3) Sliding zone of the east side of the base pit along the level: According to the detailed geological investigation data, the direction of the tectonic line is almost parallel to the excavation sideline of the station pit at a small angle, which is an outwardly inclined structural surface. Moreover, the surrounding rock is susceptible to sliding along the structural surface, and horizontal sliding may occur after the excavation of the base pit. Under the influence of the outwardly inclined structural surface and local sliding, the rock body of the east wall is easily loosened.

(4) Geological structure: The original geomorphology of the station is a tectonically denuded flat hilly landscape. The ground elevation along the line is 228~260 m, with a height difference of about 32 m. The inclination of the rock layer is 280°~290°, with a dip angle of 65°~70°. The layers are poorly combined, especially at the junction of sandstone and sandy mudstone, where the thin layer mudification phenomenon occurs.

Sandstone is thinly mudded at the junction with sandy mudstone, and no faults have been identified along the line. The rock body is subjected to relatively weak stress, the fractures in the bedrock are more developed, and the rock body has a block structure, mainly composed of sandstone and sandy mudstone of varying thickness.

(5) Hydrological situation: The groundwater in the site area is categorised into loose layer pore water and rock fracture water. The terrain is high in the east and low in the west and has a large slope, which is not conducive to the storage of groundwater. As a result, the groundwater recharge comes primarily from atmospheric precipitation. The surface water and groundwater in the area are slightly corrosive to steel and concrete structures, making it necessary to take adequate drainage measures.

### Numerical model calculation

**Fig 3:**
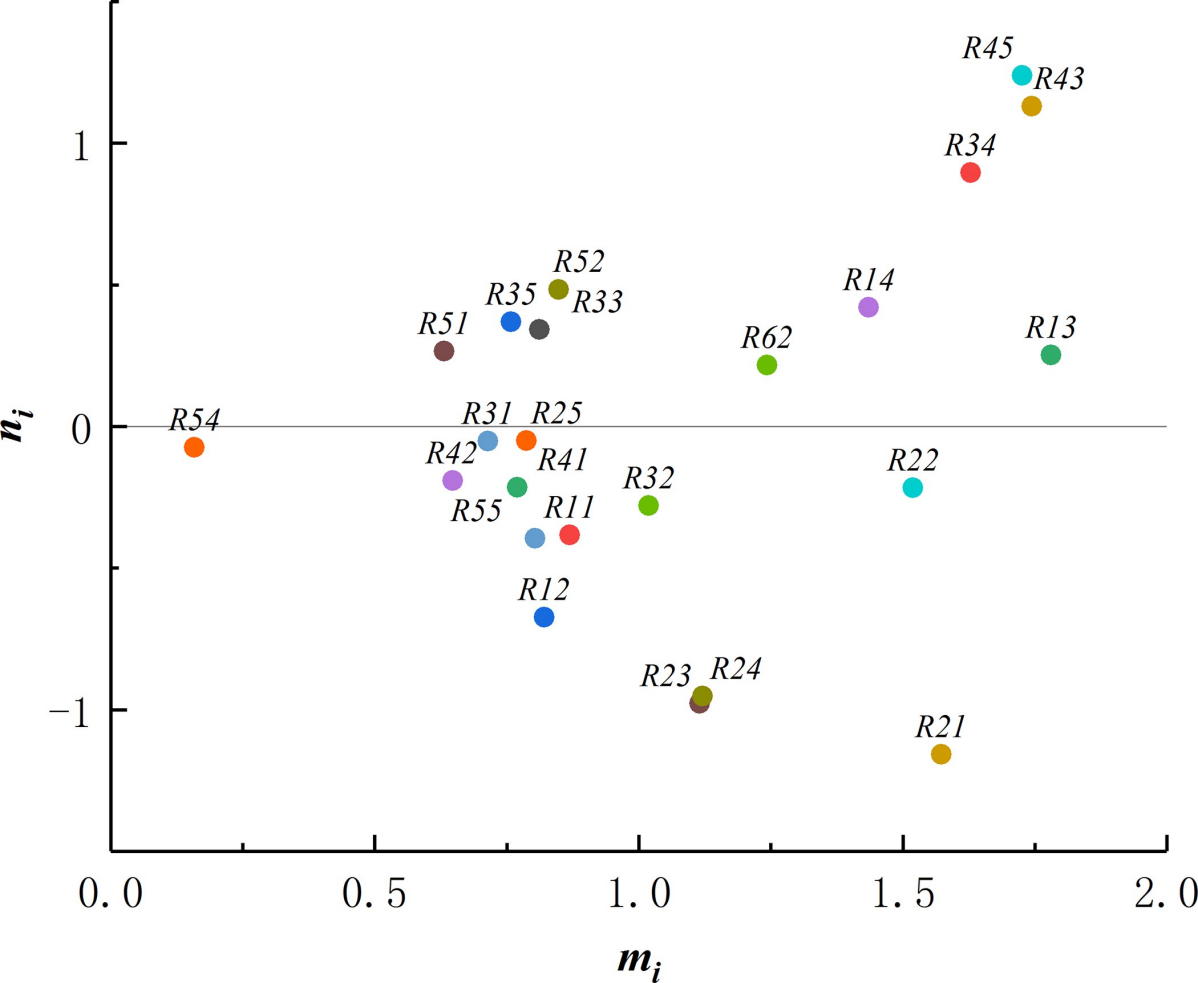
Distribution of causality-centrality.

Six experts in related fields from design institutes, construction institutes and universities were invited to evaluate the interactions between the underlying pit construction risk indicators based on the values of the grey semantic scale in Table 2. Since the risk indicators R23, R47, R53 are the same, R24 and R44 are the same, R25 and R46 are the same, and R32 and R61 are the same, only R23 is retained for the calculation of R24, R25, and R32. Different weights were given to the experts in terms of title, working years, and the number of mine construction projects, with weights for experts 1 to 6 being [0.3, 0.5], [0.4, 0.7], [0.7, 1.0], [0.5, 0.9], [0.5, 0.9], and [0.4, 0.7], respectively. According to Eq (1) to Eq (5), the direct influence matrix S is calculated, as shown in Table 4. The comprehensive influence matrix Z is calculated by Eq (6) and Eq (7), as shown in Table 5. According to Eq (8) and Eq (9), the centrality and causality of the influencing factors are calculated, as shown in Table 6. On this basis, the causal diagram of risk factors influencing the deep foundation pit construction is plotted, as shown in Fig 3.

**Table 4:** Direct impact matrix *S*.

	R11	R12	R13	R14	R21	R22	R23	R24	R25	R31	R32	R33	R34	R35	R41	R42	R43	R45	R51	R52	R54	R55	R62
**R11**	0.000	0.000	0.230	0.323	0.358	0.429	0.000	0.000	0.177	0.000	0.196	0.429	0.429	0.447	0.000	0.000	0.119	0.305	0.230	0.358	0.000	0.177	0.358
**R12**	0.000	0.000	0.230	0.461	0.305	0.097	0.262	0.000	0.213	0.196	0.263	0.119	0.323	0.429	0.251	0.000	0.251	0.377	0.415	0.721	0.229	0.161	0.305
**R13**	0.000	0.000	0.000	0.962	0.196	0.481	0.000	0.000	0.251	0.699	0.177	0.446	0.415	0.297	0.000	0.000	0.647	0.507	0.000	0.358	0.059	0.000	0.305
**R14**	0.000	0.000	0.358	0.000	0.000	0.377	0.000	0.338	0.204	0.447	0.007	0.230	0.377	0.097	0.000	0.000	0.619	0.323	0.000	0.000	0.000	0.000	0.305
**R21**	0.305	0.358	0.670	0.507	0.000	0.962	0.059	0.000	0.533	0.251	0.339	0.358	0.504	0.559	0.446	0.446	0.358	0.640	0.523	0.619	0.079	0.779	0.779
**R22**	0.305	0.000	0.801	0.667	0.196	0.000	0.000	0.000	0.270	0.251	0.000	0.230	0.392	0.196	0.000	0.000	0.377	0.464	0.721	0.613	0.000	0.263	0.808
**R23**	0.000	0.000	0.801	0.377	0.000	0.323	0.000	0.251	0.613	0.377	0.196	0.251	0.475	0.230	0.584	0.323	0.694	0.487	0.487	0.962	0.000	0.311	0.196
**R24**	0.262	0.338	0.962	0.461	0.230	0.619	0.000	0.000	0.186	0.000	0.000	0.000	0.690	0.305	0.305	0.358	0.615	0.907	0.230	0.230	0.000	0.119	0.642
**R25**	0.000	0.000	0.487	0.377	0.000	0.251	0.079	0.000	0.000	0.000	0.011	0.097	0.357	0.119	0.000	0.000	0.277	0.297	0.251	0.305	0.000	0.000	0.230
**R31**	0.000	0.000	0.429	0.119	0.000	0.177	0.000	0.000	0.000	0.000	0.000	0.000	0.059	0.000	0.000	0.338	0.962	0.637	0.000	0.000	0.000	0.000	0.196
**R32**	0.377	0.000	0.429	0.358	0.377	0.377	0.000	0.000	0.000	0.000	0.000	0.555	0.251	0.365	0.000	0.000	0.429	0.305	0.358	0.230	0.000	0.000	0.305
**R33**	0.000	0.000	0.000	0.000	0.000	0.000	0.000	0.000	0.000	0.000	0.000	0.000	0.721	0.447	0.000	0.000	0.487	0.323	0.000	0.000	0.000	0.000	0.000
**R34**	0.177	0.000	0.305	0.305	0.000	0.000	0.000	0.000	0.000	0.000	0.000	0.251	0.000	0.230	0.000	0.000	0.665	0.478	0.000	0.000	0.000	0.000	0.429
**R35**	0.000	0.000	0.177	0.230	0.000	0.000	0.000	0.000	0.000	0.000	0.000	0.059	0.177	0.000	0.000	0.000	0.721	0.161	0.000	0.000	0.000	0.000	0.000
**R41**	0.000	0.000	0.230	0.196	0.000	0.000	0.230	0.000	0.186	0.000	0.000	0.000	0.598	0.000	0.000	0.251	0.962	0.962	0.000	0.000	0.000	0.000	0.196
**R42**	0.000	0.000	0.196	0.230	0.000	0.000	0.000	0.000	0.000	0.000	0.000	0.000	0.598	0.000	0.000	0.000	0.962	0.962	0.000	0.358	0.000	0.000	0.119
**R43**	0.000	0.000	0.305	0.196	0.000	0.230	0.000	0.000	0.000	0.000	0.000	0.000	0.598	0.000	0.000	0.000	0.000	0.801	0.000	0.000	0.000	0.000	0.177
**R45**	0.000	0.000	0.196	0.305	0.000	0.000	0.000	0.000	0.000	0.000	0.000	0.311	0.598	0.000	0.262	0.000	0.196	0.000	0.000	0.000	0.000	0.000	0.000
**R51**	0.000	0.000	0.000	0.000	0.000	0.000	0.000	0.000	0.000	0.000	0.000	0.000	0.435	0.000	0.000	0.311	0.000	0.311	0.000	0.481	0.000	0.000	0.000
**R52**	0.000	0.000	0.377	0.000	0.000	0.000	0.000	0.000	0.000	0.000	0.000	0.000	0.311	0.000	0.000	0.000	0.409	0.292	0.000	0.000	0.000	0.000	0.000
**R54**	0.000	0.000	0.000	0.000	0.000	0.000	0.000	0.000	0.000	0.000	0.000	0.000	0.453	0.000	0.000	0.000	0.177	0.338	0.000	0.000	0.000	0.000	0.000
**R55**	0.119	0.000	0.526	0.177	0.000	0.251	0.000	0.000	0.165	0.000	0.691	0.000	0.338	0.000	0.507	0.000	0.717	0.481	0.119	0.338	0.000	0.000	0.000
**R62**	0.251	0.000	0.000	0.262	0.000	0.358	0.000	0.000	0.165	0.000	0.962	0.507	0.000	0.507	0.000	0.000	0.000	0.533	0.338	0.000	0.000	0.000	0.000

**Table 5:** Integrated impact matrix *Z*.

	R11	R12	R13	R14	R21	R22	R23	R24	R25	R31	R32	R33	R34	R35	R41	R42	R43	R45	R51	R52	R54	R55	R62
**R11**	0.006	0.001	0.038	0.046	0.036	0.050	0.000	0.001	0.022	0.006	0.025	0.051	0.060	0.052	0.004	0.003	0.035	0.051	0.029	0.042	0.000	0.020	0.046
**R12**	0.005	0.001	0.041	0.059	0.030	0.022	0.025	0.002	0.027	0.025	0.031	0.023	0.055	0.049	0.028	0.005	0.053	0.063	0.045	0.077	0.022	0.018	0.041
**R13**	0.005	0.001	0.022	0.105	0.020	0.057	0.000	0.003	0.029	0.072	0.022	0.053	0.063	0.038	0.003	0.003	0.090	0.076	0.008	0.040	0.006	0.003	0.044
**R14**	0.004	0.001	0.048	0.016	0.003	0.044	0.000	0.032	0.023	0.046	0.005	0.030	0.052	0.017	0.002	0.003	0.076	0.052	0.006	0.006	0.000	0.002	0.040
**R21**	0.038	0.033	0.098	0.083	0.008	0.109	0.008	0.003	0.061	0.036	0.048	0.056	0.092	0.071	0.049	0.046	0.087	0.113	0.065	0.080	0.009	0.076	0.097
**R22**	0.033	0.001	0.092	0.083	0.022	0.017	0.000	0.003	0.032	0.033	0.012	0.038	0.064	0.032	0.004	0.004	0.065	0.075	0.073	0.068	0.001	0.027	0.088
**R23**	0.005	0.001	0.102	0.062	0.004	0.046	0.002	0.025	0.064	0.045	0.025	0.038	0.081	0.033	0.058	0.035	0.108	0.090	0.052	0.102	0.001	0.030	0.037
**R24**	0.031	0.032	0.114	0.075	0.027	0.075	0.002	0.002	0.028	0.013	0.012	0.021	0.099	0.045	0.034	0.036	0.095	0.125	0.033	0.038	0.001	0.016	0.081
**R25**	0.003	0.000	0.055	0.047	0.002	0.030	0.007	0.002	0.004	0.006	0.005	0.017	0.047	0.017	0.002	0.001	0.041	0.043	0.027	0.034	0.000	0.001	0.030
**R31**	0.002	0.000	0.048	0.023	0.001	0.023	0.000	0.001	0.003	0.005	0.003	0.007	0.022	0.004	0.002	0.031	0.100	0.076	0.003	0.004	0.000	0.001	0.025
**R32**	0.039	0.001	0.055	0.050	0.038	0.047	0.000	0.002	0.007	0.008	0.007	0.063	0.047	0.046	0.003	0.003	0.062	0.052	0.040	0.032	0.001	0.005	0.042
**R33**	0.001	0.000	0.005	0.006	0.000	0.002	0.000	0.000	0.000	0.001	0.001	0.004	0.073	0.043	0.001	0.000	0.054	0.039	0.000	0.000	0.000	0.000	0.004
**R34**	0.018	0.000	0.035	0.037	0.002	0.007	0.000	0.001	0.003	0.004	0.005	0.031	0.014	0.027	0.002	0.000	0.072	0.058	0.003	0.003	0.000	0.001	0.045
**R35**	0.001	0.000	0.021	0.026	0.001	0.004	0.000	0.001	0.001	0.003	0.001	0.008	0.024	0.002	0.001	0.000	0.072	0.024	0.000	0.001	0.000	0.000	0.004
**R41**	0.002	0.000	0.034	0.032	0.001	0.007	0.021	0.001	0.020	0.004	0.003	0.009	0.074	0.005	0.004	0.024	0.105	0.109	0.003	0.005	0.000	0.001	0.027
**R42**	0.002	0.000	0.028	0.032	0.001	0.005	0.000	0.001	0.002	0.003	0.002	0.008	0.071	0.004	0.003	0.000	0.101	0.104	0.001	0.034	0.000	0.000	0.018
**R43**	0.002	0.000	0.035	0.028	0.001	0.025	0.000	0.001	0.002	0.004	0.003	0.008	0.065	0.005	0.002	0.000	0.012	0.083	0.003	0.003	0.000	0.001	0.023
**R45**	0.001	0.000	0.023	0.034	0.001	0.003	0.001	0.001	0.002	0.003	0.001	0.032	0.063	0.004	0.024	0.001	0.030	0.011	0.001	0.001	0.000	0.000	0.006
**R51**	0.001	0.000	0.005	0.004	0.000	0.001	0.000	0.000	0.000	0.000	0.000	0.003	0.046	0.001	0.001	0.029	0.009	0.036	0.000	0.045	0.000	0.000	0.003
**R52**	0.001	0.000	0.038	0.007	0.001	0.003	0.000	0.000	0.001	0.003	0.001	0.004	0.035	0.002	0.001	0.000	0.044	0.035	0.000	0.002	0.000	0.000	0.004
**R54**	0.001	0.000	0.003	0.003	0.000	0.001	0.000	0.000	0.000	0.000	0.000	0.002	0.045	0.001	0.001	0.000	0.020	0.035	0.000	0.000	0.000	0.000	0.002
**R55**	0.015	0.000	0.064	0.034	0.005	0.033	0.001	0.001	0.020	0.006	0.066	0.012	0.053	0.008	0.049	0.002	0.088	0.069	0.017	0.039	0.000	0.001	0.013
**R62**	0.028	0.000	0.013	0.037	0.005	0.041	0.000	0.001	0.018	0.003	0.090	0.058	0.019	0.056	0.002	0.001	0.018	0.064	0.038	0.008	0.000	0.002	0.010

**Table 6:** Centrality and causality of risk factors.

	Influence Degree *f*_*i*_	Influenced Degree *e*_*i*_	Centre Degree *m*_*i*_	Cause Degree *n*_*i*_
**R11**	0.243	0.626	0.869	-0.383
**R12**	0.073	0.747	0.821	-0.674
**R13**	1.017	0.764	1.781	0.253
**R14**	0.928	0.508	1.435	0.420
**R21**	0.207	1.365	1.572	-1.158
**R22**	0.651	0.868	1.519	-0.217
**R23**	0.069	1.046	1.115	-0.977
**R24**	0.084	1.036	1.120	-0.951
**R25**	0.369	0.419	0.788	-0.050
**R31**	0.331	0.383	0.714	-0.052
**R32**	0.370	0.649	1.019	-0.279
**R33**	0.577	0.235	0.812	0.343
**R34**	1.262	0.366	1.628	0.897
**R35**	0.564	0.194	0.758	0.370
**R41**	0.278	0.492	0.770	-0.214
**R42**	0.229	0.419	0.648	-0.190
**R43**	1.437	0.307	1.744	1.130
**R45**	1.482	0.244	1.726	1.238
**R51**	0.449	0.183	0.631	0.266
**R52**	0.666	0.183	0.849	0.483
**R54**	0.042	0.117	0.159	-0.074
**R55**	0.205	0.599	0.804	-0.394
**R62**	0.730	0.513	1.243	0.217

### Analysis and recommendations

(1) Cause factor analysis. According to Table 6, a cause degree greater than 0 for a risk indicator suggests that the risk indicator has a greater influence on other indicators. The order of risk influencing factors during deep foundation pit construction from large to small is R45>R43>R34>R52>R14>R35>R33>R51>R13>R62, of which R45 (failure of horizontal support system), R43 (insufficient stiffness and strength of enclosure structure), R34 (cracking of joint), R52 (insufficient depth of enclosure structure into the ground), R14 (verticality of trench wall is not required) have higher risk levels. Cause factors mainly include support instability and enclosure structure leakage among the risk indicators. In the process of deep foundation pit construction, the horizontal support system and maintenance structure should be emphasised, and the programme design should be completed in advance. Relevant supervision and management work should be strengthened during the construction process to ensure structural stability, and the concrete should be poured and cured in strict accordance with the construction sequence, thereby avoiding cracks in the concrete. Throughout the construction process, the quality of materials and construction should be ensured to make the construction quality conform to the corresponding standards and specifications.

(2) Analysis of outcome factors. An indicator with a cause degree below 0 suggests that other risk indicators have a greater influence on this indicator as a result factor. According to the centrality and causality of influence factors in Table 6, the resultant factors of risk during deep foundation pit construction are R11, R12, R21, R22, R23, R24, R25, R31, R32, R41, R42, R54, and R55. These factors have a more significant influence and are more likely to be affected by other risk factors, thus leading to risks in deep foundation pit construction. The resultant risk factors are ranked as R25>R31>R54>R42>R41>R22>R32>R11>R55>R12>R24>R23>R21, with R25 (excessive slope steepness) being the main resultant factor susceptible to the influence of other risk factors, resulting in the risk of deep foundation pit construction. In the process of deep foundation pit construction, the deep foundation pit is characterised by large depth and large scale. As a result, slight negligence in the downward excavation process may lead to excessive slope steepness, thus triggering the risk of soil landslides or support instability in the foundation pit. Therefore, it is necessary to guarantee the dynamic implementation of detection and strengthen the safety and quality awareness of construction personnel during deep foundation pit construction.

(3) Importance analysis. The centrality indicates the role of the risk factors in the deep foundation pit construction, with a larger centrality indicating a greater role of the risk factors in constraining decision-making. According to Table 6, it can be seen that the centrality of each risk factor from the largest to the smallest is R13>R43>R45>R34>R21>R22>R14>R62>R24>R23>R32>R11>.R52>R12>R33>R55>R25>R41>R35>R31>R42>R51>R54. Among these factors, R13 (excessive lateral earth pressure on the pile wall), R43 (insufficient stiffness and strength of the enclosure structure), R45 (failure of the horizontal support system), R34 (cracking of joints), R21 (poor geology), R22 (decrease in the self-stability of the soil), R14 (verticality of the trench wall is not required). R34 (cracking of joints), R21 (poor geology), R22 (decrease in self-stability of soil), R14 (verticality of trench wall not required), R62 (destruction of rainwater well), R24 (pile load over top of the slope), R23 (over-digging), and R32 (no rainfall) are the 11 most prominent risk indicators affecting the risk of deep foundation pit construction. Therefore, these factors should be emphasised during the construction process.

### Comprehensive risk assessment and response

#### Calculation of risk factors and weights

The risk factor evaluation index system for the deep foundation pit construction is a collection of risk factors, and the weight of the primary indicators and secondary indicators are calculated according to Eq (10). Moreover, 10 experts (4 technical experts from construction institutes, 2 project managing experts from construction institutes, 2 experts from design institutes, and 2 experts from universities) are invited to score the probability of occurrence of the secondary risk indicators according to the characteristics of the deep foundation pit project. Finally, the evaluation table of risk factors for deep foundation pit construction is obtained, as shown in Table 7.

**Table 7:** Evaluation table of risk factors for deep foundation pit construction.

Type of incident	Weighting	Cause of incident	Weight	VeryHigh	High	Medium	Low	Very Low
Borehole wall collapse (R1)	0.1653	Failure of mud quality (R11)	0.0293	0	0	4	4	2
		Existence of subsurface obstructions (R12)	0.0277	0	0	5	3	2
		Excessive lateral earth pressure on pile wall (R13)	0.0600	2	2	6	0	0
		Verticality of trench wall not in accordance with requirements (R14)	0.0483	0	4	4	2	0
Landslide of soil in pit (R2)	0.2060	Poor geology (R21)	0.0530	3	3	4	0	0
		Decrease in soil stability (R22)	0.0512	0	0	6	3	1
		Excessive excavation (R23)	0.0376	0	1	4	4	1
		Excessive stockpiling at the top of the slope (R24)	0.0377	0	0	4	3	3
		Excessive slope steepness (R25)	0.0265	1	1	5	3	0
Leakage from enclosure structure (R3)	0.1661	The spinning pile diameter does not meet design requirements (R31)	0.0241	0	0	0	7	3
		Precipitation not in place (R32)	0.0343	0	2	4	4	0
		Mud trapped in joints (R33)	0.0274	1	2	4	3	0
		Joint cracking (R34)	0.0548	0	3	3	4	0
		Honeycombing and delamination of maintenance body (R35)	0.0255	0	2	3	5	0
Support instability (R4)	0.2665	Improper choice of support programme (R41)	0.0259	0	0	2	5	3
		Inadequate design of enclosure structure (R42)	0.0218	0	0	2	4	4
		Insufficient stiffness and strength of enclosure structure (R43)	0.0588	0	0	5	5	0
		Excessive pile load at the top of the embankment (R44)	0.0377	0	0	4	3	3
		Failure of horizontal support system (R45)	0.0581	0	2	2	2	4
		Excessive slope steepness (R46)	0.0265	1	1	5	3	0
		Over-excavation (R47)	0.0376	0	1	4	4	1
Enclosure kick damage (R5)	0.1199	Insufficient soil reinforcement in the pit (R51)	0.0213	0	0	2	6	2
		Insufficient depth of enclosure in soil (R52)	0.0286	0	0	0	7	3
		Over-excavation (R53)	0.0376	0	1	4	4	1
		Base plate not poured in time (R54)	0.0054	0	0	2	6	2
		Failure to design for pressurised water (R55)	0.0271	0	0	0	2	8
Surging or spillage at the bottom of the pit (R6)	0.0762	Insufficient rainfall (R61)	0.0343	0	2	4	4	0
		Destruction of rainwater wells (R62)	0.0419	0	1	3	3	3

#### Determination of the fuzzy evaluation matrix

According to Table 7, the affiliation degree of accident causes is calculated, and the fuzzy evaluation matrices are obtained as follows.

$R_{1}=[\begin{array}{c} 0.0\;0.0\;0.4\;0.4\;0.2\\ 0.0\;0.0\;0.5\;0.3\;0.2\\ 0.2\;0.2\;0.6\;0.0\;0.0\\ 0.0\;0.4\;0.4\;0.2\;0.0 \end{array}]$ $R_{2}=[\begin{array}{c} 0.3\;0.3\;0.4\;0.0\;0.0\\ 0.0\;0.0\;0.6\;0.3\;0.1\\ 0.0\;0.1\;0.4\;0.4\;0.1\\ 0.0\;0.0\;0.4\;0.3\;0.3\\ 0.1\;0.1\;0.5\;0.3\;0.0 \end{array}]$ $R_{3}=[\begin{array}{c} 0.0\;0.0\;0.0\;0.7\;0.3\\ 0.0\;0.2\;0.4\;0.4\;0.0\\ 0.1\;0.2\;0.4\;0.3\;0.0\\ 0.0\;0.3\;0.3\;0.4\;0.0\\ 0.0\;0.2\;0.3\;0.5\;0.0 \end{array}]$ $R_{4}=[\begin{array}{c} 0.0\;0.0\;0.2\;0.5\;0.3\\ 0.0\;0.0\;0.2\;0.4\;0.4\\ 0.0\;0.0\;0.5\;0.5\;0.0\\ 0.0\;0.0\;0.4\;0.3\;0.3\\ 0.0\;0.2\;0.2\;0.2\;0.4\\ 0.1\;0.1\;0.5\;0.3\;0.0\\ 0.0\;0.1\;0.4\;0.4\;0.1 \end{array}]$ $R_{5}=[\begin{array}{c} 0.0\;0.0\;0.2\;0.6\;0.2\\ 0.0\;0.0\;0.0\;0.7\;0.3\\ 0.0\;0.1\;0.4\;0.4\;0.1\\ 0.0\;0.0\;0.2\;0.6\;0.2\\ 0.0\;0.0\;0.0\;0.2\;0.8 \end{array}]$ $R_{6}=[\begin{array}{c} 0.0\;0.2\;0.4\;0.4\;0.0\\ 0.0\;0.1\;0.3\;0.3\;0.3 \end{array}]$

#### Calculation of fuzzy overall evaluation results

Based on the weight of each accident cause, the fuzzy evaluation vector $B_{i},i=1,2,3,4,5,6$. Based on Eq (14), six types of accident indicators (borehole wall collapse, pit soil landslide, enclosure leakage, support instability, enclosure kick damage, and surging or spillage at the bottom of the pit) can be calculated.

     *B*_1_ = (0.0120, 0.0313, 0.0809, 0.0297, 0.0114)

     *B*_2_ = (0.0185, 0.0223, 0.0953, 0.0497, 0.0202)

     *B*_3_ = (0.0027, 0.0339, 0.0488, 0.0735, 0.0072)

     *B*_4_ = (0.0027, 0.0180, 0.0940, 0.0970, 0.0549)

     *B*_5_ = (0.0000, 0.0038, 0.0203, 0.0564, 0.0393)

     *B*_6_ = (0.0000, 0.0111, 0.0263, 0.0263, 0.0126)

Based on the weights of the accident-type indicators, the fuzzy overall evaluation vector *C* is calculated by Es (15) and (16).

*C* = (0.0070, 0.0215, 0.0706, 0.0620, 0.0275)

#### Construction countermeasures and recommendations

According to the principle of maximum affiliation, the maximum value in the overall fuzzy comprehensive evaluation is 0.0706, indicating that the overall risk level of this deep foundation pit project is medium.Combined with the previous analysis of centrality and causality, it is necessary to focus on the factors of excessive lateral earth pressure on the pile wall, poor level of geology, and failure of the support system during the construction of this deep foundation pit project. Furthermore, risk control should be strengthened, and a comprehensive risk prevention and control plan should be formulated in advance. The contractor conducted further geological investigations based on evaluation results and developed a special maintenance structure construction programme. Emphasis was placed on monitoring the lateral earth pressure on the pile wall to ensure safety.

## Discussion

### Sensitivity analysis

In order to prevent the subjectivity of the experts from influencing the calculation results, the weights of each expert were changed sequentially, with all other conditions remaining unchanged. Afterwards, the above data were calculated using the Grey-DEMATEL method and the changes in the centrality-causality graphs were recorded for sensitivity analysis.

**Fig 4:**
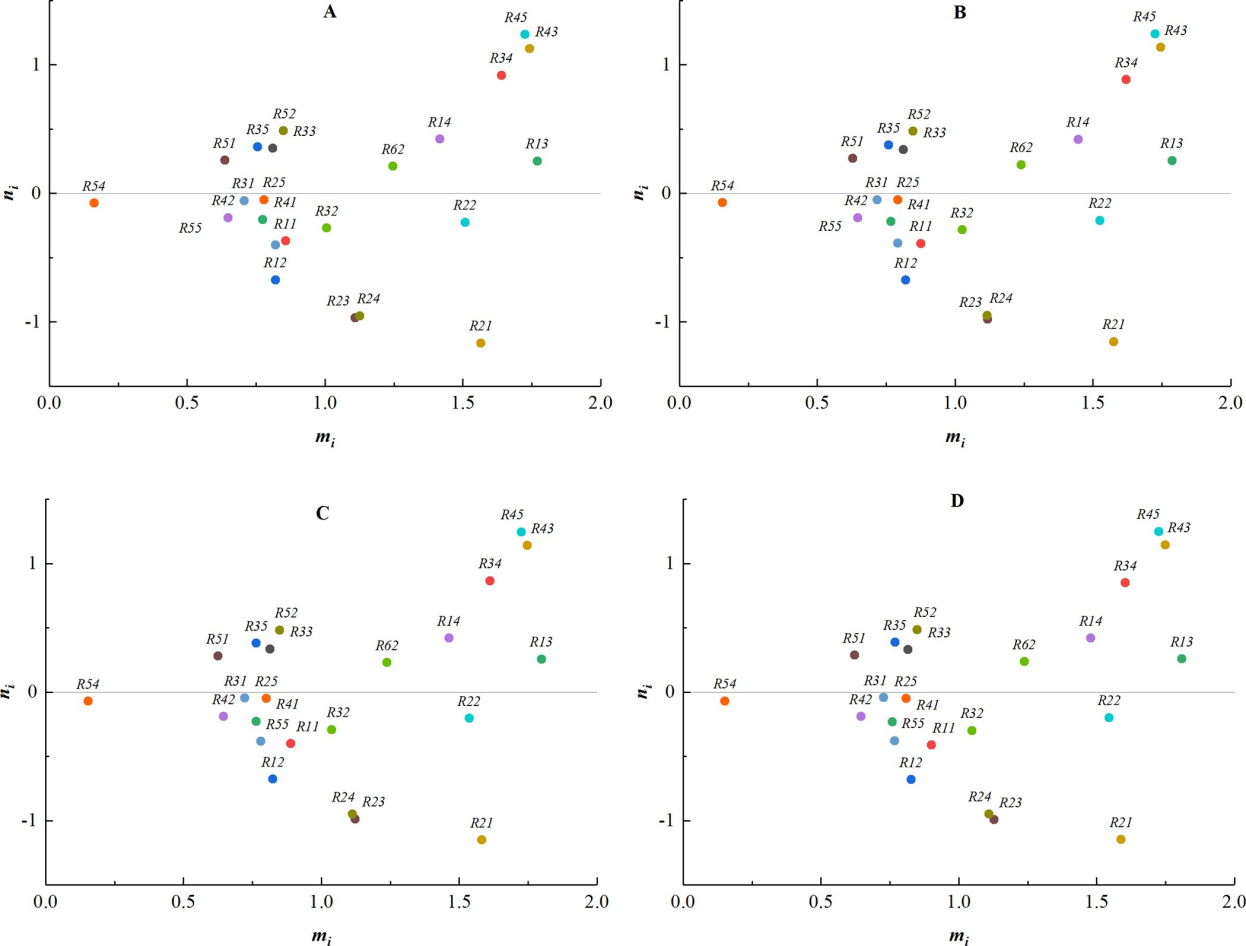
The weights of expert 1. (A) Weight [0.0, 0.3]; (B) Weight [0.4, 0.7]; (C) Weight [0.5, 0.9]; (D) Weight [0.7, 1.0].

**Fig 5:**
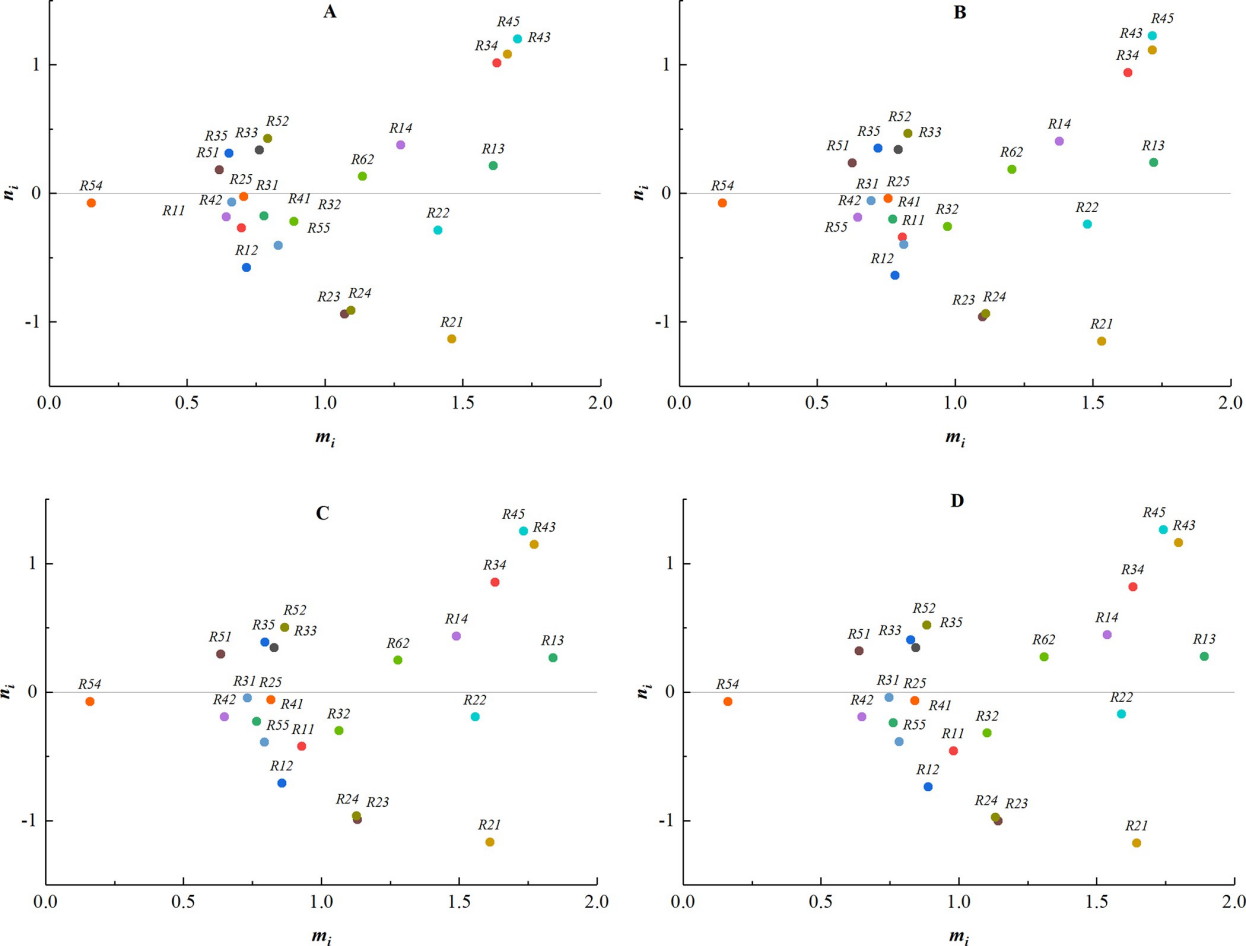
The weights of expert 2. (A) Weight [0.0, 0.3]; (B) Weight [0.3,0.5]; (C) Weight [0.5,0.9]; (D) Weight [0.7,1.0].

**Fig 6:**
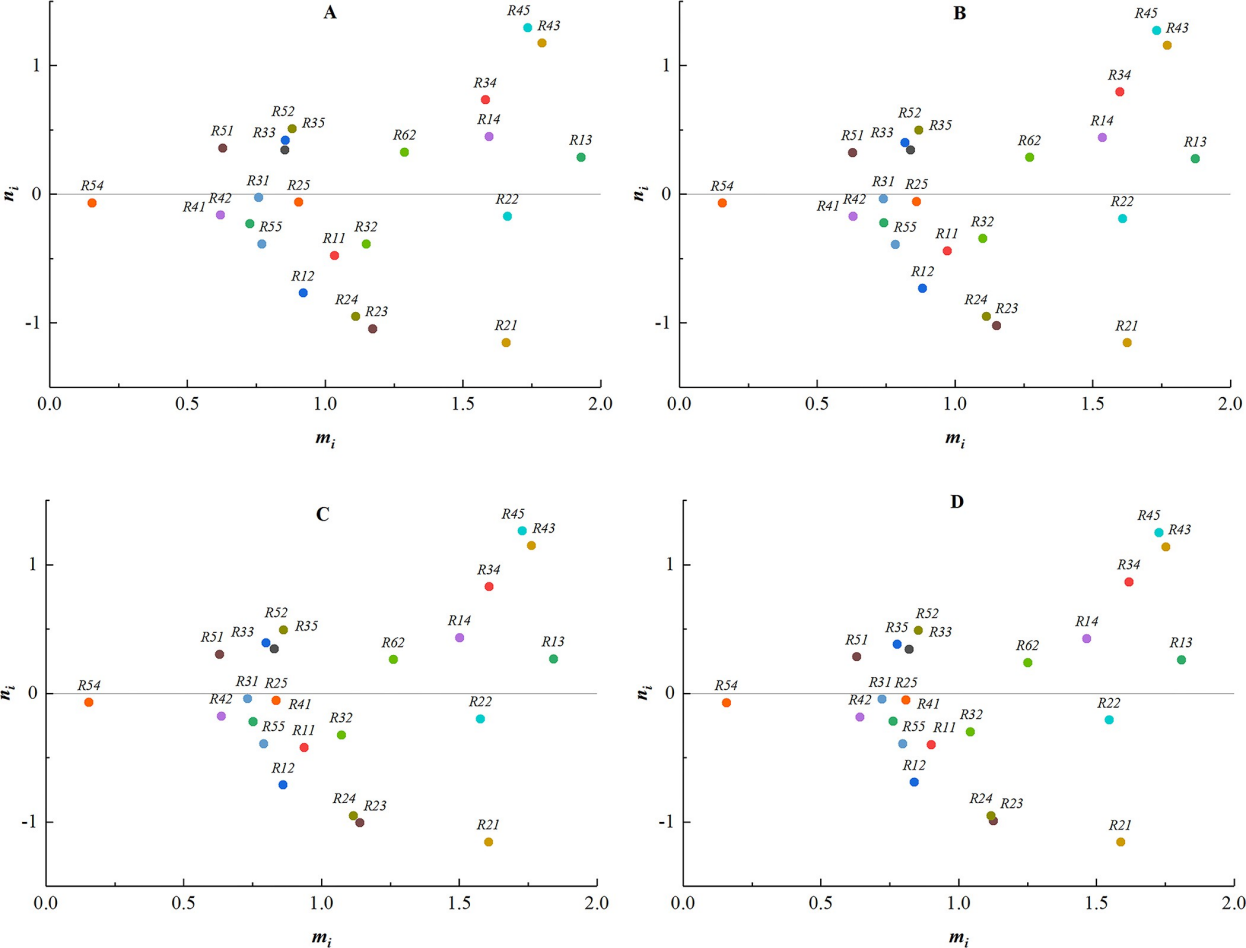
The weights of expert 3. (A) Weight [0.0, 0.3]; (B) Weight [0.3, 0.5]; (C) Weight [0.4, 0.7]; (D) Weight [0.5, 0.9].

**Fig 7:**
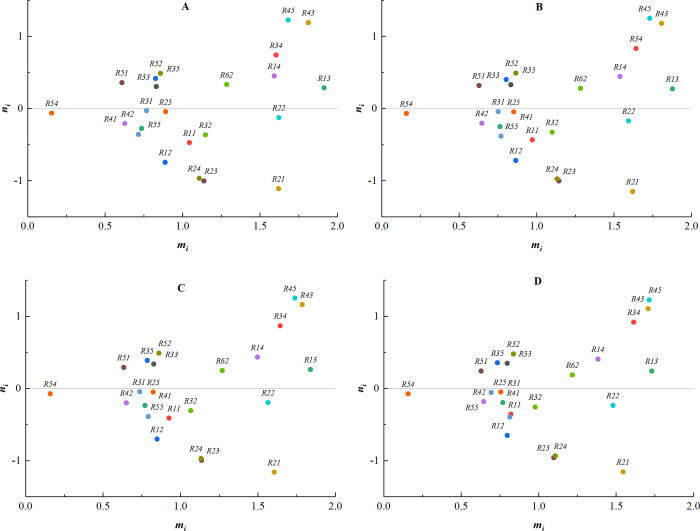
The weights of expert 4. (A) Weight [0.0, 0.3]; (B) Weight [0.3, 0.5]; (C) Weight [0.4, 0.7]; (D) Weight [0.7, 1.0].

**Fig 8:**
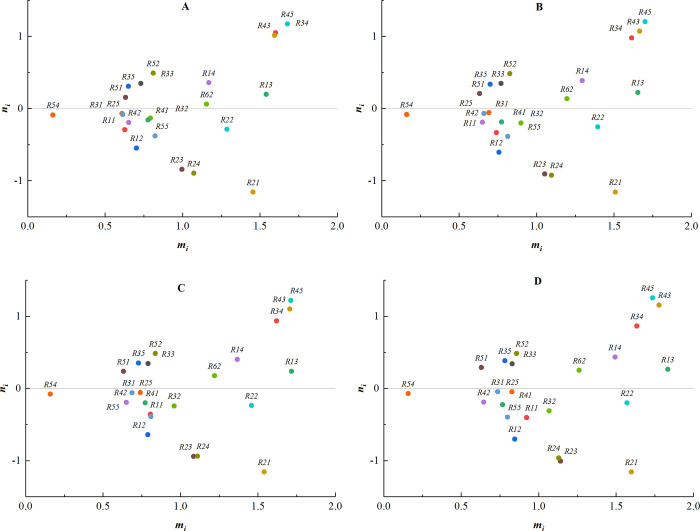
The weights of expert 5. (A) Weight [0.0, 0.3]; (B) Weight [0.3, 0.5]; (C) Weight [0.4, 0.7]; (D) Weight [0.7, 1.0].

**Fig 9:**
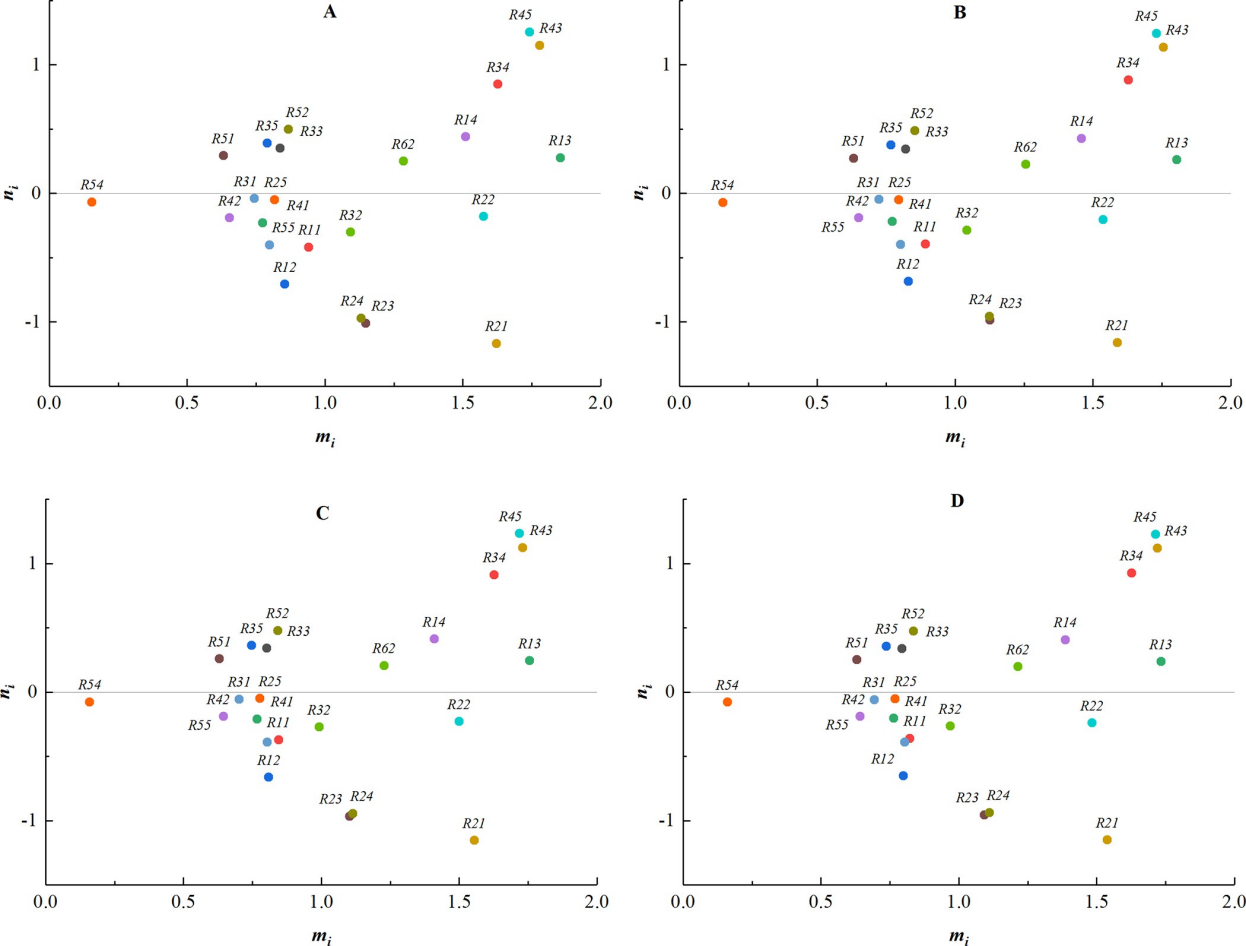
The weights of expert 6. (A) Weight [0.0, 0.3]; (B) Weight [0.3, 0.5]; (C) Weight [0.5, 0.9]; (D) Weight [0.7, 1.0].

(1) The weights of expert 1 are changed to [0.0, 0.3], [0.4, 0.7], [0.5, 0.9], [0.7, 1.0], and the corresponding centrality-causality diagrams are shown in Fig 4.

(2) The weights of expert 2 are sequentially changed to [0.0, 0.3], [0.3, 0.5], [0.5, 0.9], [0.7, 1.0], and the corresponding centrality-causality diagrams are shown in Fig 5.

(3) The weights of expert 3 are sequentially changed to [0.0, 0.3], [0.3, 0.5], [0.4, 0.7], [0.5, 0.9], and the corresponding centrality-causality diagrams are shown in Fig 6.

(4) The weights of expert 4 are sequentially changed to [0.0, 0.3], [0.3, 0.5], [0.4, 0.7], [0.7, 1.0], and the corresponding centrality-causality diagrams are shown in Fig 7.

(5) The weights of expert 5 are sequentially changed to [0.0, 0.3], [0.3, 0.5], [0.4, 0.7], [0.7, 1.0], and the corresponding centrality-causality diagrams are shown in Fig 8.

(6) The weights of expert 6 are sequentially changed to [0.0, 0.3], [0.3, 0.5], [0.5, 0.9], [0.7, 1.0], and the corresponding centrality-causality diagrams are shown in Fig 9.

As can be seen from the above centrality-causality diagrams, the order of almost all the factors remains unchanged when different weights are given to each expert. Only the values of some indicators exhibit minor changes, indicating that the size of the expert weights has less influence on the results. The negative influence of the subjective factors is basically eliminated, demonstrating the objectivity and authenticity of the results.

### Comparative analysis of methods

In order to verify the accuracy of Grey-DEMATEL-fuzzy comprehensive evaluation method, for the case of this project, on the basis of the risk evaluation level division and scoring experts are consistent, respectively, the introduction of Analytic hierarchy process(AHP) and Analytic hierarchy process—fuzzy comprehensive evaluation method of the two evaluation methods for comparative calculations [43–45], in which the evaluation level of risk influencing factors are divided into Ⅰhigh, Ⅱ higher, Ⅲ medium, Ⅳ lower, Ⅴ lower,. Corresponding to the probability of occurrence of [0.8,1], [0.6,0.8), [0.4,0.6), [0.2,0.4), [0.0,0.2), respectively, to get the results of the risk level of the different evaluation models of the deep foundation pit project are shown in Tables 8 and 9 below.

**Table 8:** Calculation results of risk levels of accident types for different evaluation models.

Evaluation models	Type of incident	Value					Risk level
AHP	R1	0.4504					Ⅲ
	R2	0.4738					Ⅲ
	R3	0.3633					Ⅳ
	R4	0.3220					Ⅳ
	R5	0.2549					Ⅳ
	R6	0.3700					Ⅳ
AHP-Fuzzy Comprehensive Evaluation Method	R1	0.0050	0.0108	**0.0424**	0.0228	0.0104	Ⅲ
	R2	0.0155	0.0244	**0.0746**	0.0489	0.0149	Ⅲ
	R3	0.0004	0.0048	0.0080	**0.0221**	0.0058	Ⅳ
	R4	0.0005	0.0016	0.0179	**0.0241**	0.0160	Ⅳ
	R5	0.0000	0.0012	0.0306	**0.1294**	0.0894	Ⅳ
	R6	0.0000	0.0473	**0.1230**	**0.1230**	0.0852	Ⅲ or Ⅳ
Grey-DEMATEL-Fuzzy Comprehensive Evaluation Method	R1	0.0120	0.0313	**0.0809**	0.0297	0.0114	Ⅲ
	R2	0.0185	0.0223	**0.0953**	0.0497	0.0202	Ⅲ
	R3	0.0027	0.0339	0.0488	**0.0735**	0.0072	Ⅳ
	R4	0.0027	0.0180	0.0940	**0.0970**	0.0549	Ⅳ
	R5	0.0000	0.0038	0.0203	**0.0564**	0.0393	Ⅳ
	R6	0.0000	0.0111	**0.0263**	0.0263	0.0126	Ⅲ

**Table 9:** Calculation of the overall risk level of the different evaluation models.

Evaluation models	Total assessed value					Overall risk level
AHP	0.3639					Ⅳ
AHP-Fuzzy Comprehensive Evaluation Method	0.0033	0.0238	0.0728	**0.0921**	0.0594	Ⅳ
Grey-DEMATEL-Fuzzy Comprehensive Evaluation Method	0.0070	0.0215	**0.0706**	0.0620	0.0275	Ⅲ

From the above calculation results, it can be obtained that: the evaluation grades calculated by the three different evaluation models are generally the same, but for the evaluation results of the type of accident of sudden surge at the bottom of the pit or flow of soil, the evaluation grade of AHP is Ⅳ, the evaluation grade of the AHP-Fuzzy Comprehensive Evaluation Method is either Ⅲ or Ⅳ, and the evaluation grade of the Grey-DEMATEL-Fuzzy Comprehensive Evaluation Method is Ⅲ; at the same time, the evaluation grade of the total risk evaluation, the evaluation grade of AHP is Ⅳ, AHP-fuzzy comprehensive evaluation method is Ⅳ, and Grey-DEMATEL-fuzzy comprehensive evaluation method is Ⅲ. According to the characteristics of the model itself, Grey-DEMATEL-fuzzy comprehensive evaluation method is relatively objective, while AHP and AHP-fuzzy comprehensive evaluation method are relatively subjective, which leads to different evaluation levels for the type of accident of the sudden surge of the bottom of the pit or the flow of soil. This leads to the emergence of different evaluation levels for the type of accident, which affects the total risk level differently. Comparing with the actual situation of the project in the construction process, the risk level of this accident type of sudden surge at the bottom of the pit or runoff is medium, and the total risk evaluation level of medium is more in line with the actual situation of the project site. Therefore, this paper adopts Grey-DEMATEL-fuzzy comprehensive evaluation method with higher practicality.

## Conclusion

Deep foundation pit construction faces many risk factors due to its deep excavation depth, large pit size, and complex geological environment characteristics. In this study, the deep foundation pit construction project of Haitangxi subway station in Chongqing is taken as an example, and the Grey-DEMATEL-Fuzzy Comprehensive Evaluation method is adopted to evaluate and analyse the risk of the deep foundation pit construction process. The conclusions of this study are as follows:

(1) Through the literature review and expert consultation, six major risk accidents in the process of deep foundation pit construction are summarised. Moreover, the causing factors of each accident are analysed through the risk decomposition structure, and 23 risk indicators are identified, forming a complete risk indicator system for deep foundation pit construction.

(2) Aiming at the lack of information and subjectivity in the risk evaluation process, the Grey-DEMATEL method is introduced to calculate and analyse each risk indicator of deep foundation pit construction. Moreover, sensitivity analysis is conducted to avoid the influence of subjective factors on the results, and a more accurate table of the centrality and cause of influencing factors is obtained. The results show that issues such as the failure of the horizontal support system, insufficient stiffness and strength of the enclosure structure, cracking of joints, insufficient depth of the enclosure structure into the ground, and failure to require verticality of the pit wall have a large influence on other risk indicators. The steepness of the slope is the indicator most susceptible to other risk factors, and excessive lateral earth pressure on the pile wall is the most prominent risk factor. If the stiffness and strength are insufficient, emphasis should be placed on preventing the failure of the horizontal support system.

(3) Based on the Grey-DEMATEL index factor analysis method, the weight of each risk factor is determined, and then the fuzzy comprehensive evaluation method is used to evaluate the overall risk level of the project. The results show that the overall risk level of the deep foundation pit construction project of Haitangxi Metro Station is medium. Finally, the evaluation results are fed back to the constructor to enable the design of a construction plan focusing on the special maintenance structure. Emphasis is also placed on solving the problem of excessive lateral earth pressure borne by the pile wall in the construction process, thus guaranteeing the safety of deep foundation pit construction.
